# Supplementary material for: Transcriptomic diversity of innate lymphoid cells in human lymph nodes compared to BM and spleen
Source: Commun Biol. 2024 Jun 25;7:769. doi: 10.1038/s42003-024-06450-9 (PMC11199704; doi:10.1038/s42003-024-06450-9)
Supplement: Supplementary file 2 — Supplementary Information [file 42003_2024_6450_MOESM2_ESM.pdf]

COMMSBIO-23-4093

Supplemental Materials

Transcriptomic Diversity of Innate Lymphoid Cells in Human Lymph Node Compared to BM and Spleen

Elaheh Hashemi[1,2], Colleen McCarthy[3], Sridhar Rao[1,5,6], and Subramaniam Malarkannan[1,2,4,5,*]

[1]Blood Research Institute, Versiti, Milwaukee, WI

[2]Department of Microbiology and Immunology, Medical College of Wisconsin (MCW)

[3]Wisconsin Organ Donor Center, Versiti, Milwaukee, WI

[4]Division of Hematology and Oncology, Department of Medicine, MCW

[5]Division of Hematology, Oncology, and Bone Marrow Transplantation, Department of Pediatrics, MCW, Milwaukee, WI

[6] Department of Cell Biology, Neurobiology, and Anatomy, MCW

Correspondence should be addressed to SM (smalarkannan@Versiti.org)

Age and gender information of tissue donors

Supplemental Table 1

Sample	Age	Gender
**BM1**	19	Female
**BM2**	25	Female
**BM3**	28	Male
**BM4**	44	Male
**LN-A1**	15	Female
**LN-A2**	53	Female
**LN-A3**	51	Male
**LN-T1**	56	Female
**LN-T2**	51	Male
**LN-T3**	60	Male
**Spl1**	51	Male
**Spl2**	56	Male
**Spl3**	54	Female
**Spl4**	53	Female

Supplemental Figure 1. Transcriptomic landscape of individual donor.

(a) Identification of transcriptomically distinct cell clusters as depicted by a UMAP of each tissue sample. Abd (abdominal) LN (lymph node), Tho (thoracic) LNs, BMs (bone marrow), and Spls (spleen) Clusters were defined by integration-based analysis on the differential expressed genes (DEGs). Total cells clustered into 8 groups.

(b) The percentage of total ILCs in LNs, BMs, and Spls using flow cytometry. An anti-CD7 antibody was used to capture all ILCs, including NK cells. T cells, B cells, and monocytes were excluded by using anti-CD19, -CD20, -CD3ε, and -CD14 antibodies. An anti-CD127 antibody was used to detect helper ILCs in each tissue.

(c) Percentage of total ILCs and helper ILCs in each tissue as shown by bar graphs, and each dot represents a donor.

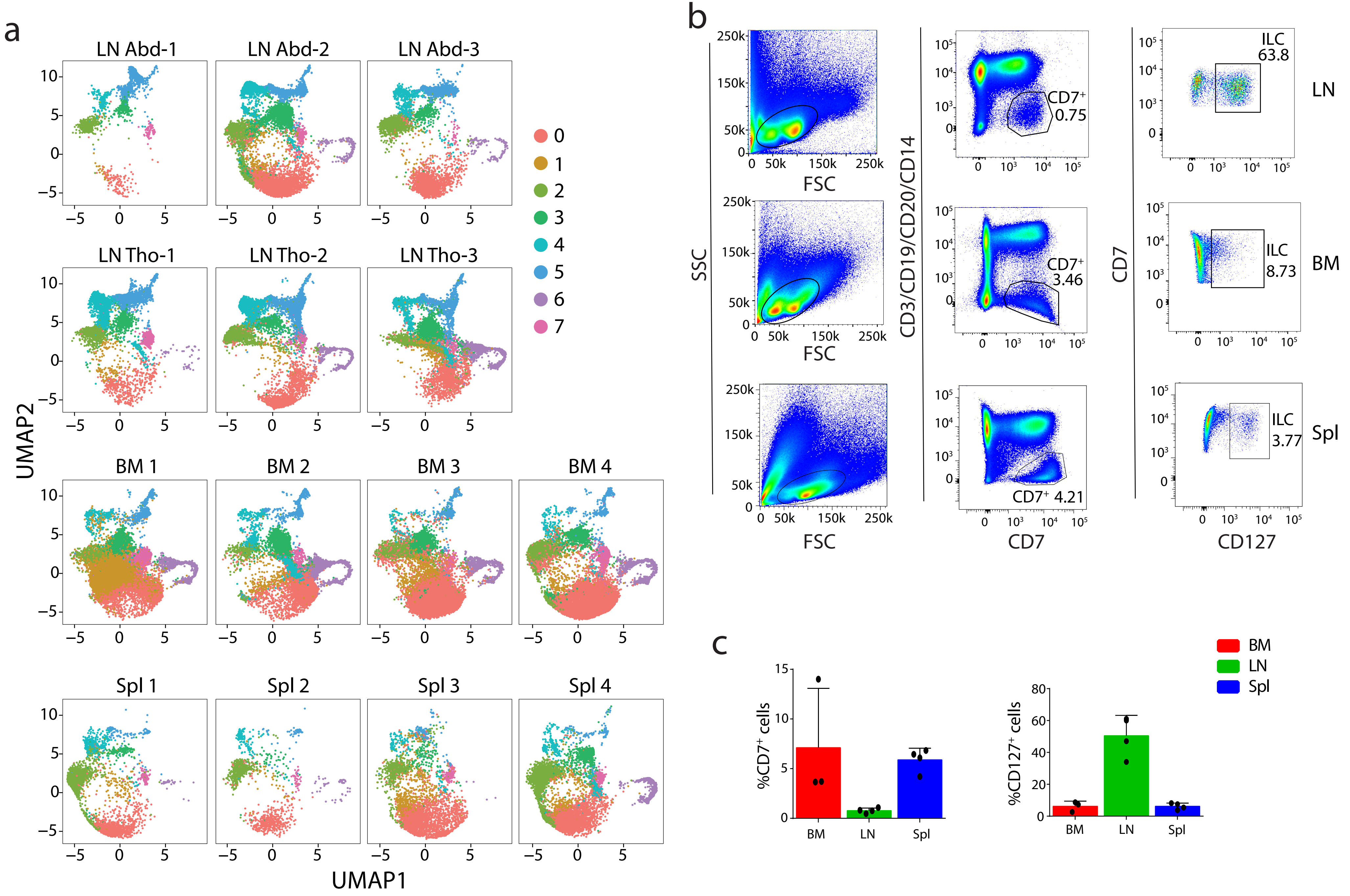
a
LN Abd-1
LN Abd-2
LN Abd-3
LN Tho-1
LN Tho-2
LN Tho-3
BM 1
BM 2
BM 3
BM 4
Spl 1
Spl 2
Spl 3
Spl 4
UMAP2
UMAP1
0
1
2
3
4
5
6
7
b
SSC
FSC
CD3/CD19/CD20/CD14
CD7
CD7+ 0.75
CD7+ 3.46
CD7+ 4.21
ILC 63.8
ILC 8.73
ILC 3.77
CD127
LN
BM
Spl
c
%CD7+ cells
%CD127+ cells
BM
LN
Spl

Supplemental Figure 2. Identify helper ILCs.

(a) The DEGs for C #4 and #5 compared to conventional NK cells visualized by heatmap. The color scale reflects Z-score from log2 fold change.

(b) Reclustering combined data from the C #4 and #5. A total 8 clusters were found after regrouping shown by UMAP.

(c) Dot plots showing the expression of known markers for canonical NK. The size of the dots shows the percentage of the cells expressing genes, and the color indicates the significance of the expression.

(d) Expression level of canonical NK gene list on the total ILCs. Module scores were calculated by the expression levels of gene lists and visualized by UMAPs. Blue to red represent levels of expression of the gene list in each feature plot.

(e) Reclustering of ILCs after removing the C #1 and #7 from the previous setting. The UMAP depicted 10 distinct clusters identified by DEGs.

(f) Dot plots showing the expression of known markers for canonical NK. The size of the dots shows the percentage of the cells expressing genes, and the color indicates the significance of the expression.

(g Expression level of canonical NK gene list on the total ILCs. Module scores were calculated by the expression levels of gene lists and visualized by UMAPs. Blue to red represent levels of expression of the gene list in each feature plot.

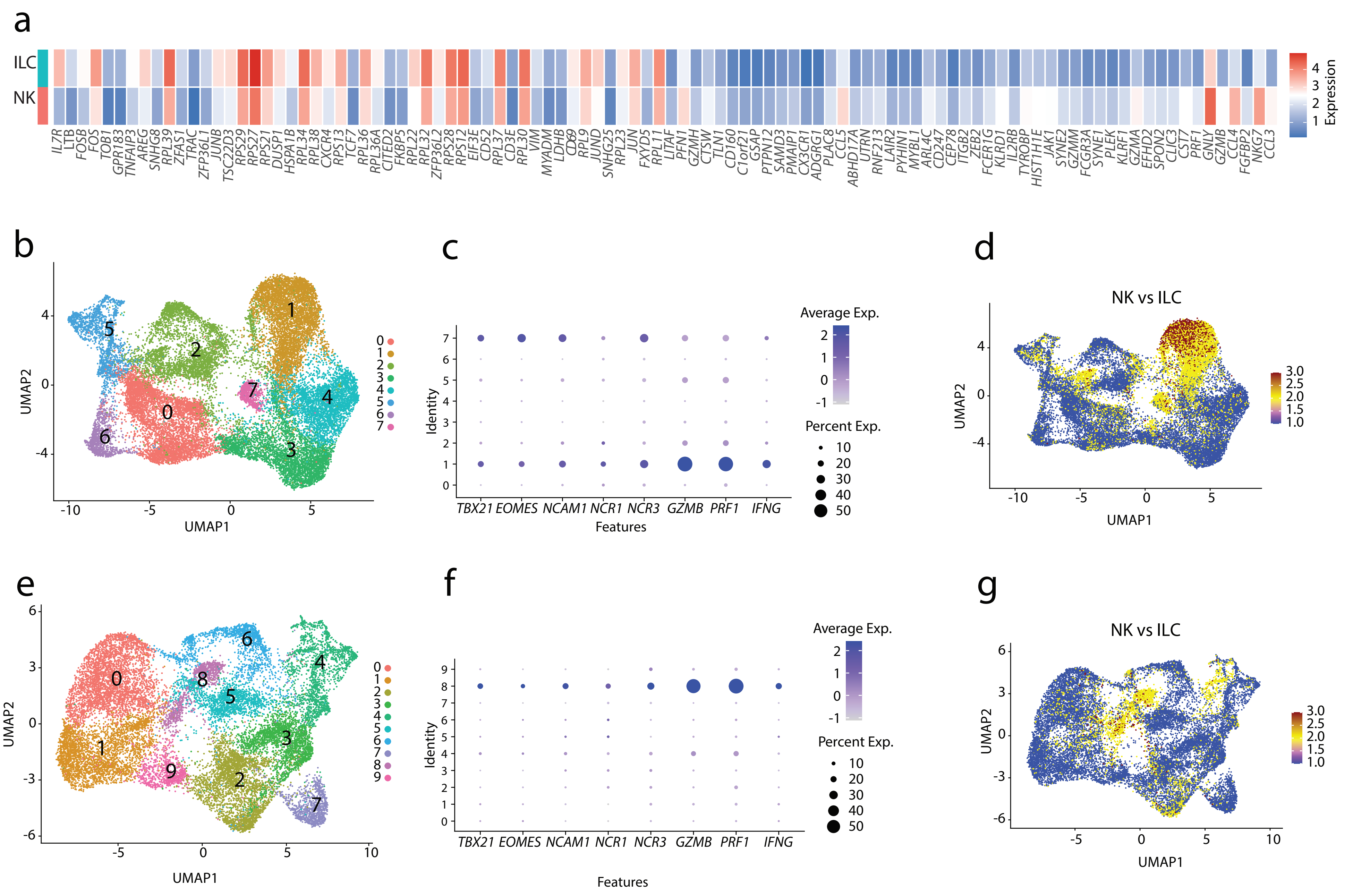
a
ILC
NK
Expression
IL7R LTB FOSB FOS TOB1 GPR183 TNFAIP3 AREG SNHG8 RPL39 ZFAS1 TRAC ZFP36L1 JUNB TSC22D3 RPS29 RPS27 RPS21 DUSP1 HSPA1B RPL34 RPL38 CXCR4 RPS13 TCF7 RPL36 RPL36A CITED2 FKBP5 RPL22 RPL32 ZFP36L2 RPS28 RPS12 EIF3E CD52 RPL37 CD3E RPL30 VIM MYADM LDHB CD69 RPL9 JUND SNHG25 RPL23 JUN FXYD5 RPL11 LITAF PFN1 GZMH CTSW TLN1 CD160 C1orf21 GSAP PTPN12 SAMD3 PMAIP1 CX3CR1 ADGRG1 PLAC8 CCL5 ABHD17A UTRN RNF213 LAIR2 PYHIN1 MYBL1 ARL4C CD247 CEP78 ITGB2 ZEB2 FCER1G KLRD1 IL2RB TYROBP HIST1H1E JAK1 SYNE2 GZMM FCGR3A SYNE1 PLEK KLRF1 GZMA EFHD2 SPON2 CLIC3 CST7 PRF1 GNLY GZMB CCL4 FGFBP2 NKG7 CCL3
b
UMAP1
UMAP2
c
Average Exp.
Percent Exp.
Identity
Features
TBX21 EOMES NCAM1 NCR1 NCR3 GZMB PRF1 IFNG
d
NK vs ILC
UMAP1
UMAP2
e
UMAP1
UMAP2
f
Average Exp.
Percent Exp.
Identity
Features
TBX21 EOMES NCAM1 NCR1 NCR3 GZMB PRF1 IFNG
g
NK vs ILC
UMAP1
UMAP2

Supplemental Figure 3. Transcriptional program and gene regulatory network (GRN) in ILCs isolated from lymph nodes.

(a) Identification of ILC subtypes in BM tissues using flow cytometry. An anti-CD7 antibody was used to capture all ILCs, including NK cells. T cells, B cells, and monocytes were excluded by using anti-CD19, -CD20, -CD3ε, and -CD14 antibodies. $CD7^{+}CD127^{+}$ cells were identified as helper ILCs. Anti-CD117 and -CRTH2 antibodies were used to identify ILC1-ILC3.

(b) The percentage of total ILCs and each subtype with each sample represented as a dot.

(c&d) Regulon activity of each TF and downstream target genes in ILC1 and ILC3 clusters is depicted by feature plots. The darker red color represents a higher level of expression. Number of genes (g) that were analyzed for each regulon in brackets.

(e) Representative transcription factors and their corresponding binding motifs for each module are shown.

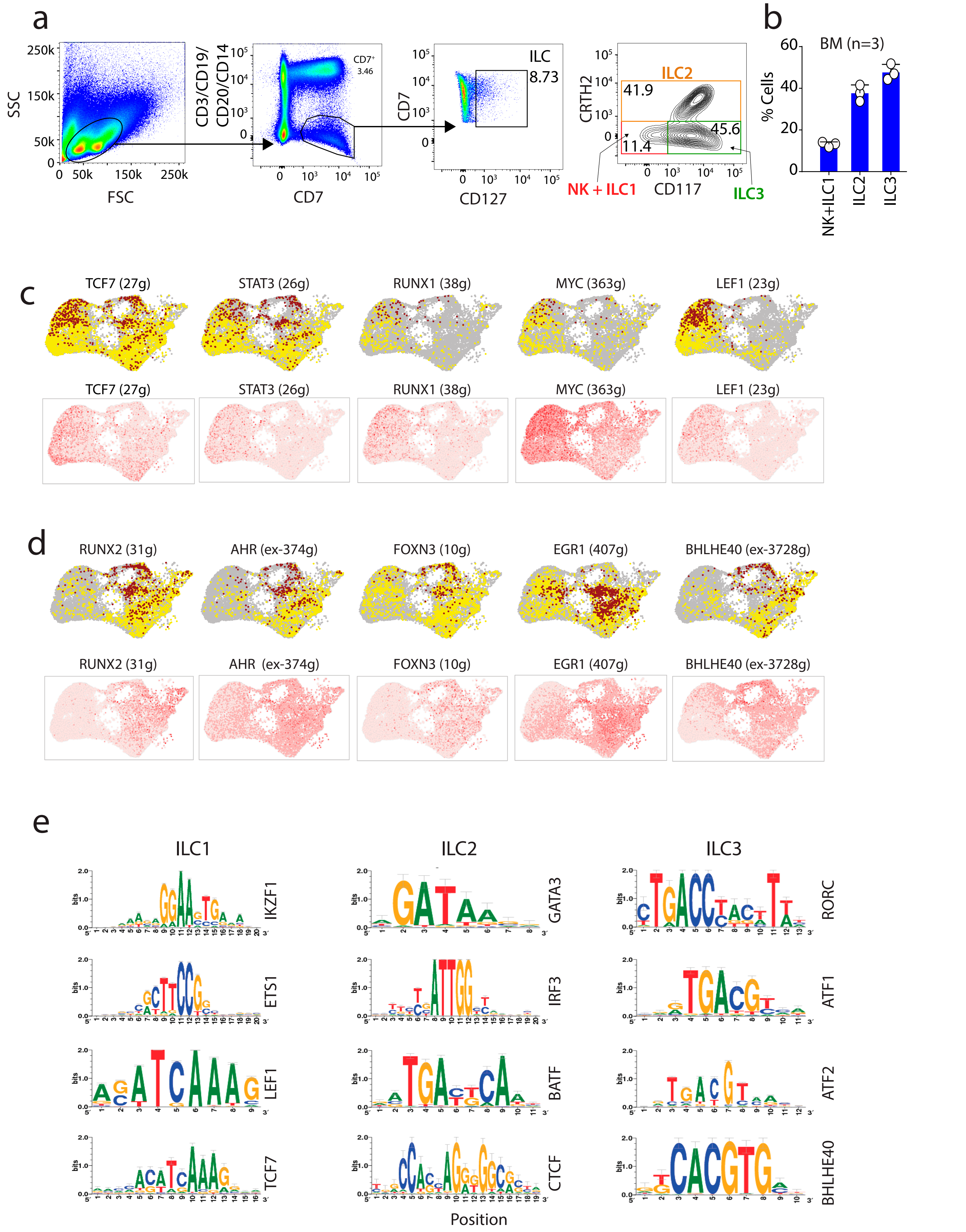
a
SSC
FSC
250k
150k
50k
0
50k
150k
250k
CD3/CD19/
CD20/CD14
CD7
CD7+
3.46
CD127
ILC
8.73
CRTH2
CD117
ILC2
41.9
45.6
11.4
NK + ILC1
ILC3
b
BM (n=3)
% Cells
60
40
20
0
NK+ILC1
ILC2
ILC3
c
TCF7 (27g)
STAT3 (26g)
RUNX1 (38g)
MYC (363g)
LEF1 (23g)
d
RUNX2 (31g)
AHR (ex-374g)
FOXN3 (10g)
EGR1 (407g)
BHLHE40 (ex-3728g)
e
ILC1
ILC2
ILC3
IKZF1
ETS1
LEF1
TCF7
GATA3
IRF3
BATF
CTCF
RORC
ATF1
ATF2
BHLHE40
bits
Position

Supplemental Figure 4. Transcriptomic landscape of progenitor population in BM and LNs.

(a) Heatmap showing the top significant DEGs in C#4 from BM and LN. Rows represent genes, and columns represent cells. Cells from the same cluster are grouped together. The color scale reflects Z-Score of log2 fold change.

(b) Heatmap showing the regulon activity of the C#4 from LN and BM color-coded by the tissues. The color bar on the top represents each tissue and related active regulons. The regulons are grouped in the color boxes based on their pattern of expression.

(c) Gene Set Enrichment Analysis (GSEA) for specific pathways in C#4 of BM and LNs. The positive running enrichment score (red line) indicates the enrichment of pathways in each group. (RLM ranked list metric; RES rank enrichment score). P-values were calculated by the Wilcoxon Rank Sum test.

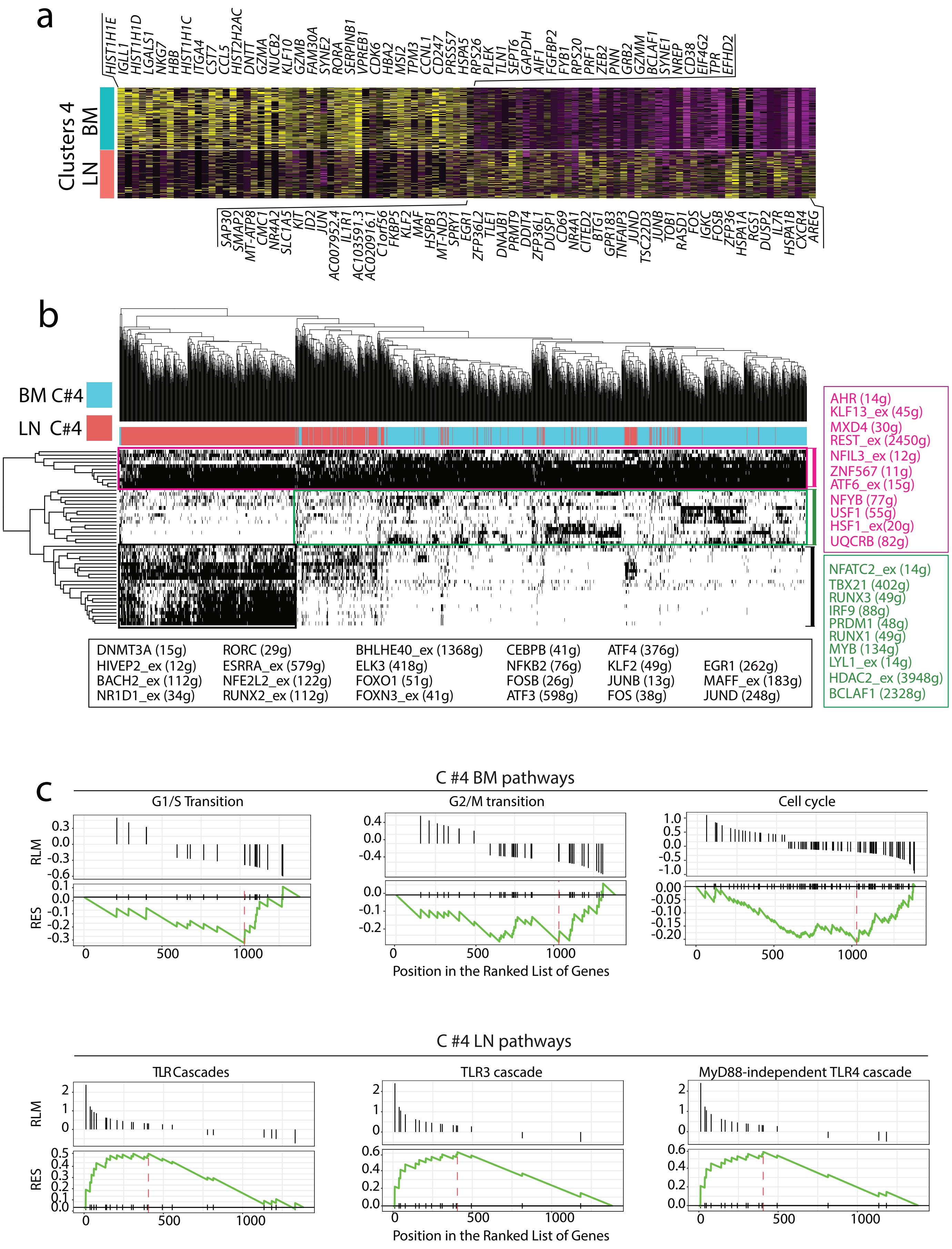
a
HIST1H1E IGLL1 HIST1H1D LGALS1 NKG7 HBB HIST1H1C ITGA4 CST7 CCL5 HIST2H2AC DNTT GZMA NUCB2 KLF10 GZMB FAM30A SYNE2 RORA SERPINB1 VPREB1 CDK6 HBA2 MSI2 TPM3 CCNL1 CD247 PRSS57 HSPA5 RPS26 PLEK TLN1 SEPT6 GAPDH AIF1 FGFBP2 FYB1 RPS20 PRF1 ZEB2 PNN GRB2 GZMM BCLAF1 SYNE1 NREP CD38 EIF4G2 TPR EFHD2
Clusters 4
BM
LN
SAP30 SMAP2 MT-ATP8 CMC1 NR4A2 SLC1A5 KIT ID2 JUN AC007952.4 IL1R1 AC103591.3 AC020916.1 C1orf56 FKBP5 KLF2 MAF HSPB1 MT-ND3 SPRY1 EGR1 ZFP36L2 TLE1 DNAJB1 PRMT9 DDIT4 ZFP36L1 DUSP1 CD69 NR4A1 CITED2 BTG1 GPR183 TNFAIP3 JUND TSC22D3 JUNB TOB1 RASD1 FOS IGKC FOSB ZFP36 HSPA1A RGS1 DUSP2 IL7R HSPA1B CXCR4 AREG
b
BM C#4
LN C#4
AHR (14g)
KLF13_ex (45g)
MXD4 (30g)
REST_ex (2450g)
NFIL3_ex (12g)
ZNF567 (11g)
ATF6_ex (15g)
NFYB (77g)
USF1 (55g)
HSF1_ex(20g)
UQCRB (82g)
NFATC2_ex (14g)
TBX21 (402g)
RUNX3 (49g)
IRF9 (88g)
PRDM1 (48g)
RUNX1 (49g)
MYB (134g)
LYL1_ex (14g)
HDAC2_ex (3948g)
BCLAF1 (2328g)
DNMT3A (15g) RORC (29g) BHLHE40_ex (1368g) CEBPB (41g) ATF4 (376g)
HIVEP2_ex (12g) ESRRA_ex (579g) ELK3 (418g) NFKB2 (76g) KLF2 (49g) EGR1 (262g)
BACH2_ex (112g) NFE2L2_ex (122g) FOXO1 (51g) FOSB (26g) JUNB (13g) MAFF_ex (183g)
NR1D1_ex (34g) RUNX2_ex (112g) FOXN3_ex (41g) ATF3 (598g) FOS (38g) JUND (248g)
c
C #4 BM pathways
G1/S Transition
G2/M transition
Cell cycle
RLM
RES
Position in the Ranked List of Genes
C #4 LN pathways
TLR Cascades
TLR3 cascade
MyD88-independent TLR4 cascade
RLM
RES
Position in the Ranked List of Genes

Supplemental Figure 5. The distinct pathways operating in progenitors and naïve ILCs in the BM and LN tissues.

(a) The barcode plots show the significantly altered GSEA pathways for C #4 in BM and C #7 LNs. The positive running enrichment score of the redline indicates the enrichment of pathways in each group. P values were calculated by the Wilcoxon Rank Sum test.

(b) RNA velocity depicting the BM progenitor development from HSC to CLP, EILP, and pILC. The black arrows indicate the direction of the ILC developments.

(c) & (d) Pseudotime analysis showing the development of nILCs to ILC1 and ILC3 using the Monocle3 package. Analysis of combined ILC1 and ILC3 with naïve ILCS indicates the potential temporal transcriptomic stage from nILCs to more mature ILCs.

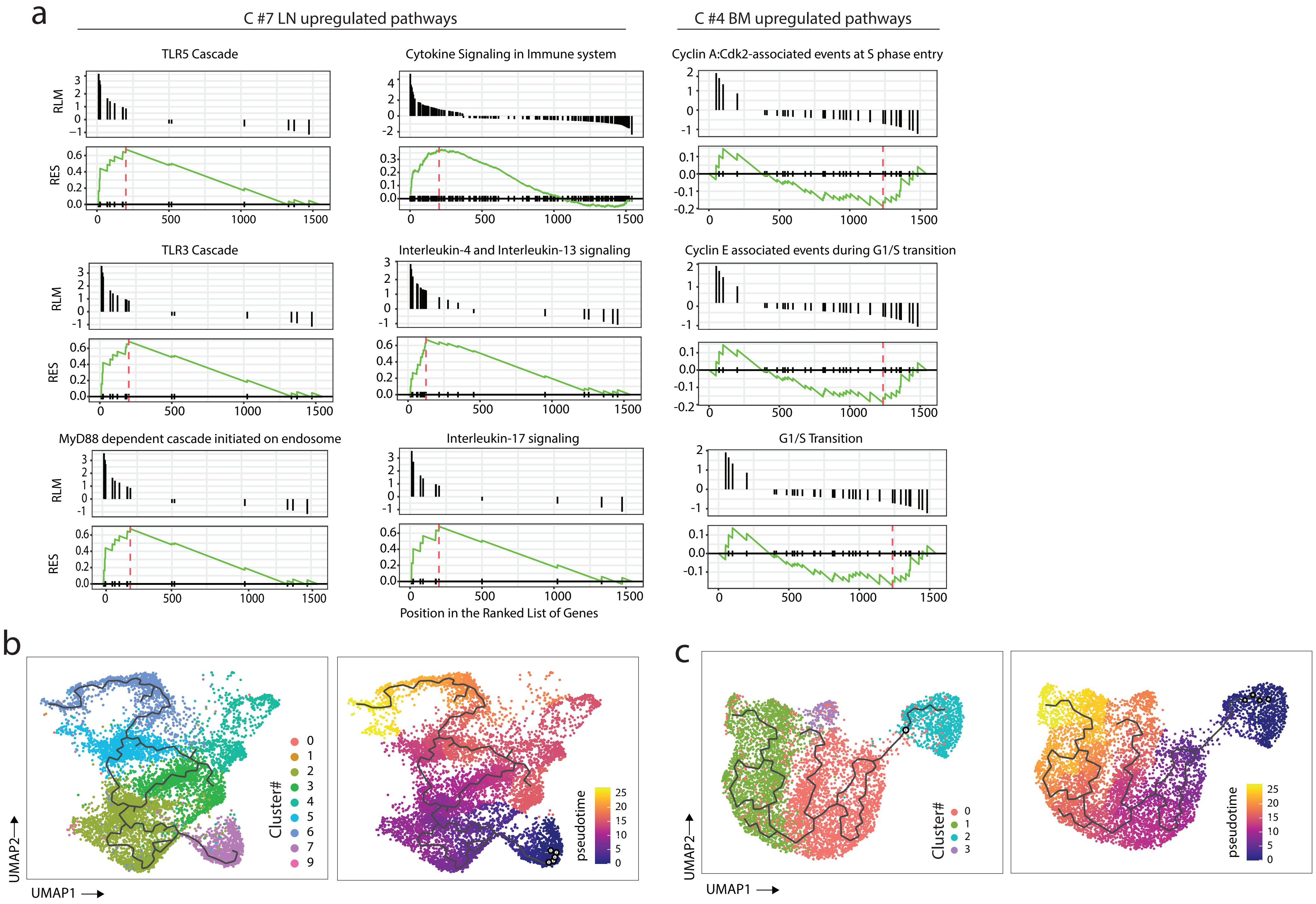
a
C #7 LN upregulated pathways
C #4 BM upregulated pathways
TLR5 Cascade
Cytokine Signaling in Immune system
Cyclin A:Cdk2-associated events at S phase entry
TLR3 Cascade
Interleukin-4 and Interleukin-13 signaling
Cyclin E associated events during G1/S transition
MyD88 dependent cascade initiated on endosome
Interleukin-17 signaling
G1/S Transition
RLM
RES
Position in the Ranked List of Genes
b
c
Cluster#
pseudotime
UMAP1
UMAP2

Supplemental Figure 6. The DEGs analysis for the Thoracic and abdominal LNs.

(a) Heatmap representing highly expressed genes in the abdominal and thoracic LNs compared to spleen and BM.

(b) Heatmap representing DEGs between human abdominal and thoracic LNs. Rows and columns represent genes and cells, respectively. The color scale reflects Z-score from log2 fold change.

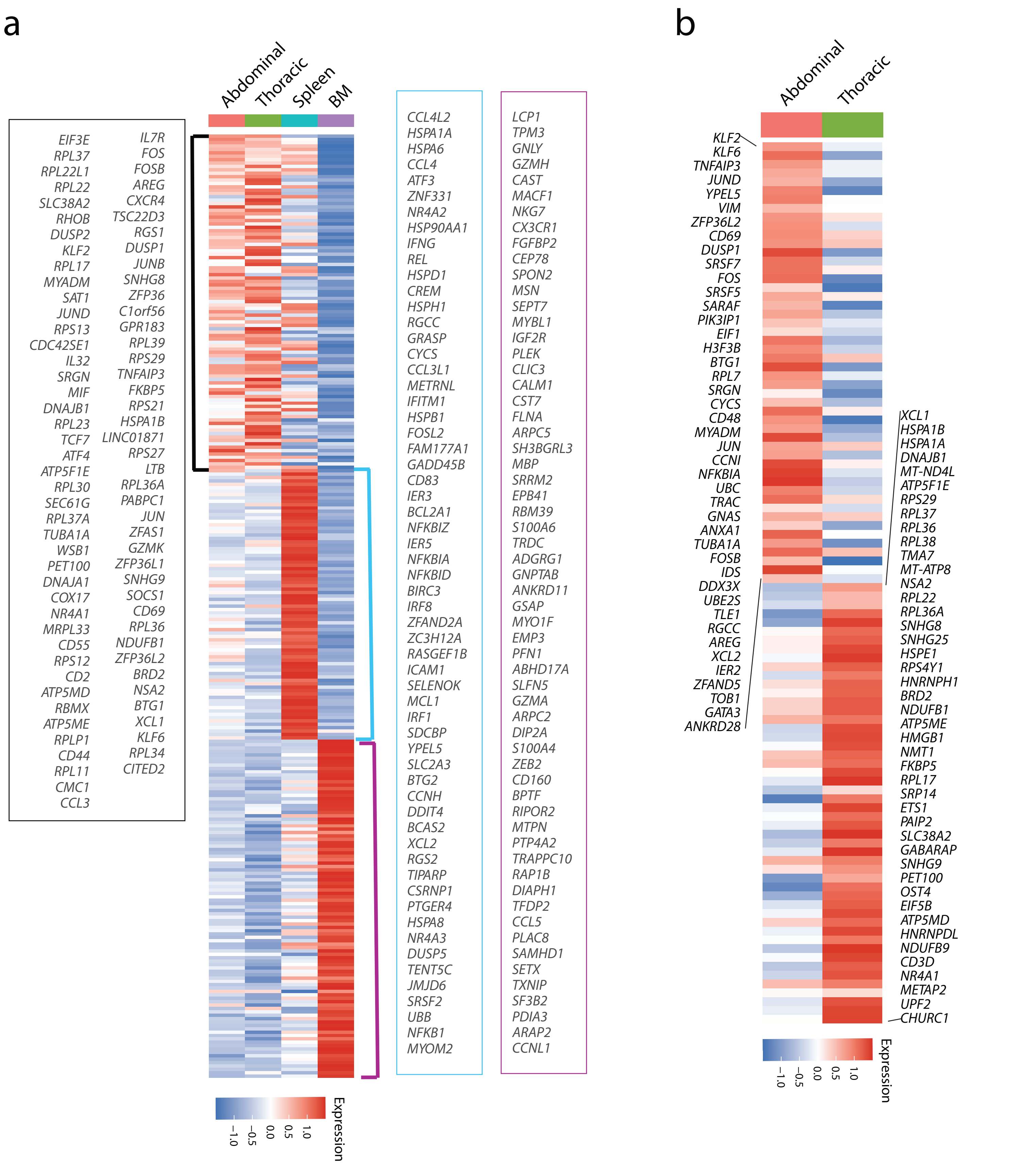
a
Abdominal
Thoracic
Spleen
BM
EIF3E IL7R
RPL37 FOS
RPL22L1 FOSB
RPL22 AREG
SLC38A2 CXCR4
RHOB TSC22D3
DUSP2 RGS1
KLF2 DUSP1
RPL17 JUNB
MYADM SNHG8
SAT1 ZFP36
JUND C1orf56
RPS13 GPR183
CDC42SE1 RPL39
IL32 RPS29
SRGN TNFAIP3
MIF FKBP5
DNAJB1 RPS21
RPL23 HSPA1B
TCF7 LINC01871
ATF4 RPS27
ATP5F1E LTB
RPL30 RPL36A
SEC61G PABPC1
RPL37A JUN
TUBA1A ZFAS1
WSB1 GZMK
PET100 ZFP36L1
DNAJA1 SNHG9
COX17 SOCS1
NR4A1 CD69
MRPL33 RPL36
CD55 NDUFB1
RPS12 ZFP36L2
CD2 BRD2
ATP5MD NSA2
RBMX BTG1
ATP5ME XCL1
RPLP1 KLF6
CD44 RPL34
RPL11 CITED2
CMC1
CCL3
CCL4L2
HSPA1A
HSPA6
CCL4
ATF3
ZNF331
NR4A2
HSP90AA1
IFNG
REL
HSPD1
CREM
HSPH1
RGCC
GRASP
CYCS
CCL3L1
METRNL
IFITM1
HSPB1
FOSL2
FAM177A1
GADD45B
CD83
IER3
BCL2A1
NFKBIZ
IER5
NFKBIA
NFKBID
BIRC3
IRF8
ZFAND2A
ZC3H12A
RASGEF1B
ICAM1
SELENOK
MCL1
IRF1
SDCBP
YPEL5
SLC2A3
BTG2
CCNH
DDIT4
BCAS2
XCL2
RGS2
TIPARP
CSRNP1
PTGER4
HSPA8
NR4A3
DUSP5
TENT5C
JMJD6
SRSF2
UBB
NFKB1
MYOM2
LCP1
TPM3
GNLY
GZMH
CAST
MACF1
NKG7
CX3CR1
FGFBP2
CEP78
SPON2
MSN
SEPT7
MYBL1
IGF2R
PLEK
CLIC3
CALM1
CST7
FLNA
ARPC5
SH3BGRL3
MBP
SRRM2
EPB41
RBM39
S100A6
TRDC
ADGRG1
GNPTAB
ANKRD11
GSAP
MYO1F
EMP3
PFN1
ABHD17A
SLFN5
GZMA
ARPC2
DIP2A
S100A4
ZEB2
CD160
BPTF
RIPOR2
MTPN
PTP4A2
TRAPPC10
RAP1B
DIAPH1
TFDP2
CCL5
PLAC8
SAMHD1
SETX
TXNIP
SF3B2
PDIA3
ARAP2
CCNL1
−1.0 −0.5 0.0 0.5 1.0
Expression
b
Abdominal
Thoracic
KLF2
KLF6
TNFAIP3
JUND
YPEL5
VIM
ZFP36L2
CD69
DUSP1
SRSF7
FOS
SRSF5
SARAF
PIK3IP1
EIF1
H3F3B
BTG1
RPL7
SRGN
CYCS
CD48
MYADM
JUN
CCNI
NFKBIA
UBC
TRAC
GNAS
ANXA1
TUBA1A
FOSB
IDS
DDX3X
UBE2S
TLE1
RGCC
AREG
XCL2
IER2
ZFAND5
TOB1
GATA3
ANKRD28
XCL1
HSPA1B
HSPA1A
DNAJB1
MT-ND4L
ATP5F1E
RPS29
RPL37
RPL36
RPL38
TMA7
MT-ATP8
NSA2
RPL22
RPL36A
SNHG8
SNHG25
HSPE1
RPS4Y1
HNRNPH1
BRD2
NDUFB1
ATP5ME
HMGB1
NMT1
FKBP5
RPL17
SRP14
ETS1
PAIP2
SLC38A2
GABARAP
SNHG9
PET100
OST4
EIF5B
ATP5MD
HNRNPDL
NDUFB9
CD3D
NR4A1
METAP2
UPF2
CHURC1
−1.0 −0.5 0.0 0.5 1.0
Expression

Supplemental Figure 7. The branch point analysis of ILC3 subsets development.

(a) Each box shows selected genes for the modules that are highly expressed in the ILC3 subtypes. (Modules #12,18 for C#0, module #2,7 for C#1, module #15 for C#2, and module #9 for C#3)

(b) The feature plots showing the gene module expression across pseudotime representing C#2 and C#3 and the branch point (BP). The starting point of the pseudotime trajectory is shown by #1followed by two major branch points (BPs), indicating two distinct cell fate (C_F) decisions.

(c) Gene expression of selected genes overlayed onto pseudotime trajectories of CF and BP, demonstrating the effectorness gradient of gene expression in the transition.

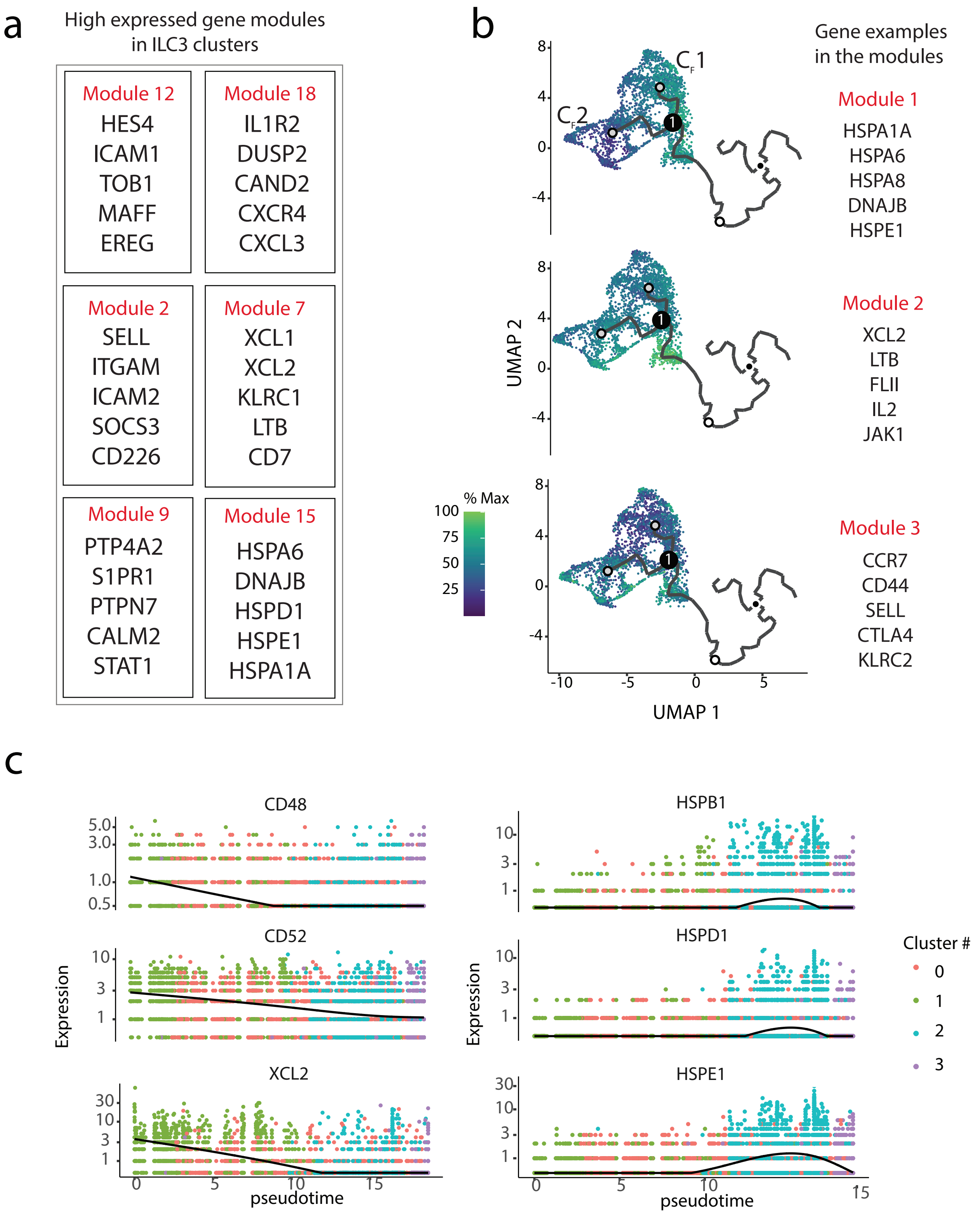
a
High expressed gene modules
in ILC3 clusters
Module 12
HES4
ICAM1
TOB1
MAFF
EREG
Module 18
IL1R2
DUSP2
CAND2
CXCR4
CXCL3
Module 2
SELL
ITGAM
ICAM2
SOCS3
CD226
Module 7
XCL1
XCL2
KLRC1
LTB
CD7
Module 9
PTP4A2
S1PR1
PTPN7
CALM2
STAT1
Module 15
HSPA6
DNAJB
HSPD1
HSPE1
HSPA1A
b
Gene examples
in the modules
Module 1
HSPA1A
HSPA6
HSPA8
DNAJB
HSPE1
Module 2
XCL2
LTB
FLII
IL2
JAK1
Module 3
CCR7
CD44
SELL
CTLA4
KLRC2
% Max
100
75
50
25
UMAP 2
UMAP 1
c
CD48
CD52
XCL2
HSPB1
HSPD1
HSPE1
Expression
pseudotime
Cluster #
0
1
2
3
