# Supplementary material for: Transcriptomic diversity of innate lymphoid cells in human lymph nodes compared to BM and spleen
Source: Commun Biol. 2024 Jun 25;7:769. doi: 10.1038/s42003-024-06450-9 (PMC11199704; doi:10.1038/s42003-024-06450-9)
Supplement: Supplementary file 3 — Reporting Summary [file 42003_2024_6450_MOESM3_ESM.pdf]

nature portfolio

Corresponding author(s): Subrammanian malarkannan

Last updated by author(s): 08.30.2023

Reporting Summary

Nature Portfolio wishes to improve the reproducibility of the work that we publish. This form provides structure for consistency and transparency in reporting. For further information on Nature Portfolio policies, see our Editorial Policies and the Editorial Policy Checklist.

Statistics

For all statistical analyses, confirm that the following items are present in the figure legend, table legend, main text, or Methods section.

n/a	Confirmed	
☐	☒	The exact sample size (n) for each experimental group/condition, given as a discrete number and unit of measurement
☐	☒	A statement on whether measurements were taken from distinct samples or whether the same sample was measured repeatedly
☐	☒	The statistical test(s) used AND whether they are one- or two-sided<br>*Only common tests should be described solely by name; describe more complex techniques in the Methods section.*
☐	☒	A description of all covariates tested
☐	☒	A description of any assumptions or corrections, such as tests of normality and adjustment for multiple comparisons
☒	☐	A full description of the statistical parameters including central tendency (e.g. means) or other basic estimates (e.g. regression coefficient) AND variation (e.g. standard deviation) or associated estimates of uncertainty (e.g. confidence intervals)
☐	☒	For null hypothesis testing, the test statistic (e.g. F, t, r) with confidence intervals, effect sizes, degrees of freedom and P value noted<br>*Give P values as exact values whenever suitable.*
☒	☐	For Bayesian analysis, information on the choice of priors and Markov chain Monte Carlo settings
☒	☐	For hierarchical and complex designs, identification of the appropriate level for tests and full reporting of outcomes
☒	☐	Estimates of effect sizes (e.g. Cohen's d, Pearson's r), indicating how they were calculated

Our web collection on statistics for biologists contains articles on many of the points above.

Software and code

Policy information about availability of computer code

Data collection	Cells were loaded on the 10X Chromium machine (10X Genomics) at target capture numbers of 8000 viable cells/sample. Library construction was performed using the 10X Genomic Chromium Single Cell 3' Reagent Kit v3.1 per manufacturer's protocols. Single-cell cDNA libraries were sequenced via Illumina Novaseq 6000 (Illumina) to a depth of around 50 million reads per cell. Raw data from each sample were demultiplex continued with the analyzing the data.
Data analysis	In this study we used software and packages that require for single-cell RNA sequencing analysis listed as bellow:<br>- Most of the analysis were conducted in R (v3.5.1 or higher)<br>- Data were aligned using NCBI Human Reference Genome Build GRCh38-3.0.0 reference genome and UMI counts quantified using 10X Genomics CellRanger software v3.0.0 using default parameters.<br>- SC RNA seq analysis including, clustering, visualization, were used the codes available in Seurat package (v 3.2.2)<br>- (GSEA)using the Reactome database was performed by the gsePathway function in the ReactomePA package v1.34.021.<br>- Pseudotime analysis was performed using Monocle 3 package (https://cole-trapnell-lab.github.io/monocle3/)<br>- RNA velocity was performed using velocyto (http://velocyto.org/) and scVelo (https://scvelo.readthedocs.io/en/stable/) packages.

For manuscripts utilizing custom algorithms or software that are central to the research but not yet described in published literature, software must be made available to editors and reviewers. We strongly encourage code deposition in a community repository (e.g. GitHub). See the Nature Portfolio guidelines for submitting code & software for further information.

Data

Policy information about availability of data

All manuscripts must include a data availability statement. This statement should provide the following information, where applicable:

- Accession codes, unique identifiers, or web links for publicly available datasets
- A description of any restrictions on data availability
- For clinical datasets or third party data, please ensure that the statement adheres to our policy

We have uploaded our all raw and analyzed data in GEO with the #GSE243033

Research involving human participants, their data, or biological material

Policy information about studies with human participants or human data. See also policy information about sex, gender (identity/presentation), and sexual orientation and race, ethnicity and racism.

Reporting on sex and gender	- 2 LN thoracic samples were males and 1 is female. 3 LN Abdominal samples were males.<br>- Spleen samples contained 2 males and 2 females<br>- Bone marrow samples also were 2 males and 2 females
Reporting on race, ethnicity, or other socially relevant groupings	There is no information about the race or ethnicity of the samples.
Population characteristics	All the samples were collected from 20-60 years old adults with not specific disease conditon or genotype.
Recruitment	The samples were collected from unknown people so no specific selection were conducted. There was a list of criteria that was exclude samples collections for the study, including Cancer or viral or bacterial acute infection.
Ethics oversight	The use of these human materials was approved by the IRB of the Versiti, Milwaukee, WI.

Note that full information on the approval of the study protocol must also be provided in the manuscript.

Field-specific reporting

Please select the one below that is the best fit for your research. If you are not sure, read the appropriate sections before making your selection.

☒ Life sciences ☐ Behavioural & social sciences ☐ Ecological, evolutionary & environmental sciences

For a reference copy of the document with all sections, see nature.com/documents/nr-reporting-summary-flat.pdf

Life sciences study design

All studies must disclose on these points even when the disclosure is negative.

Sample size	In this study we used 14 samples total. 3 abdominal lymph nodes, 3 thoracic LN, 4 BM and 4 Spleens. The reason that we choose these numbers are that 3 samples are the minimum number that we can have confidence and take out the outliers if there are any. Although the number of cells captured after first analysis of sequencing were different, but the number of samples were comparable and have at least 3 samples for each condition.
Data exclusions	There was no sample that excluded from our study.
Replication	We did not have replication. Since the samples are human samples and hard to access, so having the replication was not considered in this study.
Randomization	We did not randomized the samples, Since the samples in our study are human samples and we want to capture as many cells as we can gather for sequencing. In the analysis there are steps for excluding the cells with low quality.
Blinding	?

Reporting for specific materials, systems and methods

We require information from authors about some types of materials, experimental systems and methods used in many studies. Here, indicate whether each material, system or method listed is relevant to your study. If you are not sure if a list item applies to your research, read the appropriate section before selecting a response.

Materials & experimental systems

n/a	Involved in the study
☐	☒ Antibodies
☒	☐ Eukaryotic cell lines
☒	☐ Palaeontology and archaeology
☒	☐ Animals and other organisms
☒	☐ Clinical data
☒	☐ Dual use research of concern
☒	☐ Plants

Methods

n/a	Involved in the study
☒	☐ ChIP-seq
☐	☒ Flow cytometry
☒	☐ MRI-based neuroimaging

Antibodies

Antibodies used

CD3E (UCHT1, 300417, @ 1:100), CD19 (HIB19, 302224, @ 1:100), CD14 (HCD14, 325616, @ 1:100), CD20 (2H7, 302320, @ 1:100), CD7 (CD7-6B7, 343108, @ 1:100), CD117 (104D2, 313213, @ 1:100), CRTH2 (BM16, 350105, @ 1:100) were from BioLegend (San Diego, CA).

Validation

The antibodies are from BioLegend company with their own validations. Here are some references for each antibody based on the manufacture website:
CD117 :(Omer OS, et al. 2020. Methods Mol Biol. 2121:199. PubMed
Yi W, et al. 2021. Cell Reports. 34(13):108922. PubMed)
CRTH2: (Halim TYF et al. 2018. Immunity. 48(6):1195-1207 . PubMed
Vanoni G, et al. 2021. eLife. 10:00. PubMed)
CD7: (Bi Y, et al. 2020. Cell Reports. 30(11):3917-3931. PubMed
Yang C, et al. 2019. Nat Commun. 10:3931. PubMed)
CD20: (Leach SM, et al. 2020. Cell Rep. 33:108337. PubMed
Lovelace SE, et al. 2022. iScience. 25:105067. PubMed)
CD14: (Jackson-Jones LH, et al. 2020. Immunity. 52:700. PubMed
Liisborg C, et al. 2022. PLoS One. 17:e0269960. PubMed)
CD19: (Yang Z, et al. 2018. Front Immunol. 9:2613. PubMed
Queckborner S, et al. 2020. Stem Cell Res Ther. 11:15. PubMed)
CD3E: (Japp AS, et al. 2021. Cell. 184(3):827-839.e14. PubMed
Alter G, et al. 2020. Cell. 183(1):185-196.e14. PubMed)

Flow Cytometry

Plots

Confirm that:

☒ The axis labels state the marker and fluorochrome used (e.g. CD4-FITC).

☒ The axis scales are clearly visible. Include numbers along axes only for bottom left plot of group (a 'group' is an analysis of identical markers).

☒ All plots are contour plots with outliers or pseudocolor plots.

☒ A numerical value for number of cells or percentage (with statistics) is provided.

Methodology

Sample preparation

Mononuclear cells were washed with cold PBS + 0.4% bovine serum albumin (Sigma-Aldrich) and counted using a hemocytometer. Viability was assessed using trypan blue exclusion. Cells were incubated with Ab with the mentioned ratio for 20 minutes in fridge and then cells were washed twice with FACS buffer and follow with assessing with the flow cytometry.

Instrument

Flow cytometry analyses were conducted in LSR-II (BD Biosciences, San Jose, CA)

Software

FlowJo software (FlowJo LLC, Ashland, OR)

Cell population abundance

Among all the gated lymphocytes, the CD7+ cells were % 0.5-1 of the total lymphocyte populations. Then among the CD7+ cells, the ILC population percentage was %50-60 and then we gated on the IL7R+ cells. Among this population, as shown in the figures, majority of the cells were ILC3 then, ILC1+NK and the lower number of cells were ILC2.

Gating strategy

Lymphocyte were gated based on the low FSC and SSC as shown in the figures. For some populations, positive and negative are discreet like CD127+ cells. For the populations with more overlapped the positive and negative populations are defined with the negative controls.

☒ Tick this box to confirm that a figure exemplifying the gating strategy is provided in the Supplementary Information.
